# Human umbilical cord mesenchymal stem cell-derived exosomal miR-146a-5p reduces microglial-mediated neuroinflammation via suppression of the IRAK1/TRAF6 signaling pathway after ischemic stroke

**DOI:** 10.18632/aging.202466

**Published:** 2021-01-21

**Authors:** Zhongfei Zhang, Xiaoxiong Zou, Run Zhang, Yu Xie, Zhiming Feng, Feng Li, Jianbang Han, Haitao Sun, Qian Ouyang, Shiting Hua, Bingke Lv, Tian Hua, Zhizheng Liu, Yingqian Cai, Yuxi Zou, Yanping Tang, Xiaodan Jiang

**Affiliations:** 1 Department of Neurosurgery, Zhujiang Hospital, Southern Medical University, The National Key Clinical Specialty, The Engineering Technology Research Center of Education Ministry of China, Guangdong Provincial Key Laboratory on Brain Function Repair and Regeneration, Guangzhou, China; 2 Key Laboratory of Mental Health of the Ministry of Education, Guangdong-Hong Kong-Macao Greater Bay Area Center for Brain Science and Brain-Inspired Intelligence, Southern Medical University, Guangzhou, China

**Keywords:** mesenchymal stem/stromal cell, ischemic stroke, exosomes, neuroinflammation, microRNA

## Abstract

To investigate the therapeutic mechanism of action of transplanted stem cells and develop exosome-based nanotherapeutics for ischemic stroke, we assessed the effect of exosomes (Exos) produced by human umbilical cord mesenchymal stem cells (hUMSCs) on microglia-mediated neuroinflammation after ischemic stroke. Our results found that injected hUMSC-Exos were able to access the site of ischemic damage and could be internalized by cells both *in vivo* and *in vitro*. *In vitro*, treatment with hUMSC-Exos attenuated microglia-mediated inflammation after oxygen-glucose deprivation (OGD). *In vivo* results demonstrated that treatment with hUMSC-Exos significantly reduced infarct volume, attenuated behavioral deficits, and ameliorated microglia activation, as measured three days post-transient brain ischemia. Furthermore, miR-146a-5p knockdown (miR-146a-5p k/d Exos) partially reversed the neuroprotective effect of hUMSC-Exos. Our mechanistic study demonstrated that miR-146a-5p in hUMSC-Exos reduces microglial-mediated neuroinflammatory response through IRAK1/TRAF6 pathway. We conclude that miR-146a-5p derived from hUMSC-Exos can attenuate microglia-mediated neuroinflammation and consequent neural deficits following ischemic stroke. These results elucidate a potential therapeutic mechanism of action of mesenchymal stem cells and provide evidence that hUMSC-Exos represent a potential cell-free therapeutic option for ischemic stroke.

## INTRODUCTION

Ischemic stroke is a common cause of severe disability and death worldwide [1]. To reduce primary injury due to acute ischemic stroke and limit infarct size, timely reperfusion therapy (thrombolysis or thrombectomy) is required [2]. However, post-ischemic reperfusion itself causes damage and dysfunction in a process known as cerebral ischemia-reperfusion (I/R) injury [3, 4]. In this process, reperfusion triggers an inflammatory cascade [5], a key mechanism contributing to secondary neuronal damage and death following the initial ischemic episode [6, 7]. Overzealous up-regulation of endogenous neuroinflammatory processes leads to destruction of hypoxic tissue, induction of apoptosis, and initiation of an inflammatory cascade feed-forward loop that can enlarge the damaged region [8–10].

Microglia, the major central nervous system resident innate immune cells, play an important role in modulating neuroinflammation [11]. Cerebral IR-activated microglia undergo polarization into either “classically activated” M1 or “alternately activated” M2 phenotypes [12, 13]. While M1 microglia produce a large number of pro-inflammatory factors (e.g. IL-6, TNF-α, and IL-1β) [14–16] that impede post-injury neural regeneration and produce poorer long­term neurological outcomes [17], M2 microglia are able to perform efferocytosis and produce numerous protective and trophic factors that promote neurogenesis [18]. Decreasing microglial-mediated neuroinflammatory injury by targeting M1/M2 polarization may therefore have therapeutic potential in ischemic stroke.

Our previous meta-analysis revealed that mesenchymal stem cell (MSC) transplantation after ischemic stroke significantly improves neurological deficits and quality of life [19]. In addition, our previous original research demonstrated that MSCs are able to protect brain tissue, regulate inflammatory responses after traumatic brain injury [20] and stroke [21, 22], and can regulate microglia-mediated inflammatory responses [23]. However, the mechanisms by which MSCs modulate microglia-mediated inflammation remain unclear. For the above reasons, elucidation of these mechanisms would be of significant benefit.

Relative to human bone marrow-derived MSCs (BMSCs), human umbilical cord MSCs (hUMSCs) are more readily obtained, exhibit superior viability, are compatible with therapeutic methods featuring higher levels of patient acceptability and compliance, and are not susceptible to immune-mediated graft rejection [24, 25]. In addition, although hUMSCs are considered more primitive than bone marrow derived MSCs, they do not induce teratomas, but do exhibit immunomodulatory capabilities [26]. Stroke occurs frequently at 45-65 years old, and there is an autologous bone marrow aging problem. In clinical research, it can avoid pain during bone marrow extraction and enhance volunteer compliance. We therefore selected hUMSCs for use in the current research.

Although information regarding MSC therapeutic mechanisms of action is limited and fewer than 1% of transplanted MSCs reach and engraft at target sites (since most become entrapped in the pulmonary capillary bed due to their large size) [27], their therapeutic effect is nonetheless frequently observed [28], currently mainly attributed to MSC-produced paracrine factors. We reasoned that transplanted MSC-derived exosomes (Exos) generated *in vivo* may be one factor contributing to the distal paracrine therapeutic effects of stem cell therapy.

Small non-coding microRNAs (miRNAs) can act as inhibitors of mRNA transcription and protein translation in most cell types [29]. Cell-derived exosomes contain numerous miRNAs that are able to act both locally and - via entering circulation - at distal sites [30, 31]. Exosomes are also internalized by neighboring or distal cells, thereby modulating the function of these recipient cells [32–34]. A recent study demonstrated that MSC-derived exosome content member miR-146a decreases inflammation and enhances anti-sepsis therapeutic efficacy [35]. Our sequencing data demonstrate that hUMSC-derived exosomes contain large amounts of miR-146a-5p. We thus hypothesized that hUMSC-derived exosomes (hUMSC-Exos), via provision of miR-146a-5p to microglia and consequent regulation of microglial gene expression, decrease microglia-mediated inflammation in the ischemic mouse brain. We injected wild-type and miR-146a-5p knockdown (miR-146a-5p k/d) hUMSC-Exos into ischemic mice to test this hypothesis as well as to explore mechanisms of potential therapeutic activity.

## RESULTS

### Characterization of hUMSCs and hUMSC-Exos confirms hUMSC phenotype and demonstrates typical exosomal features

**Figure 1:**
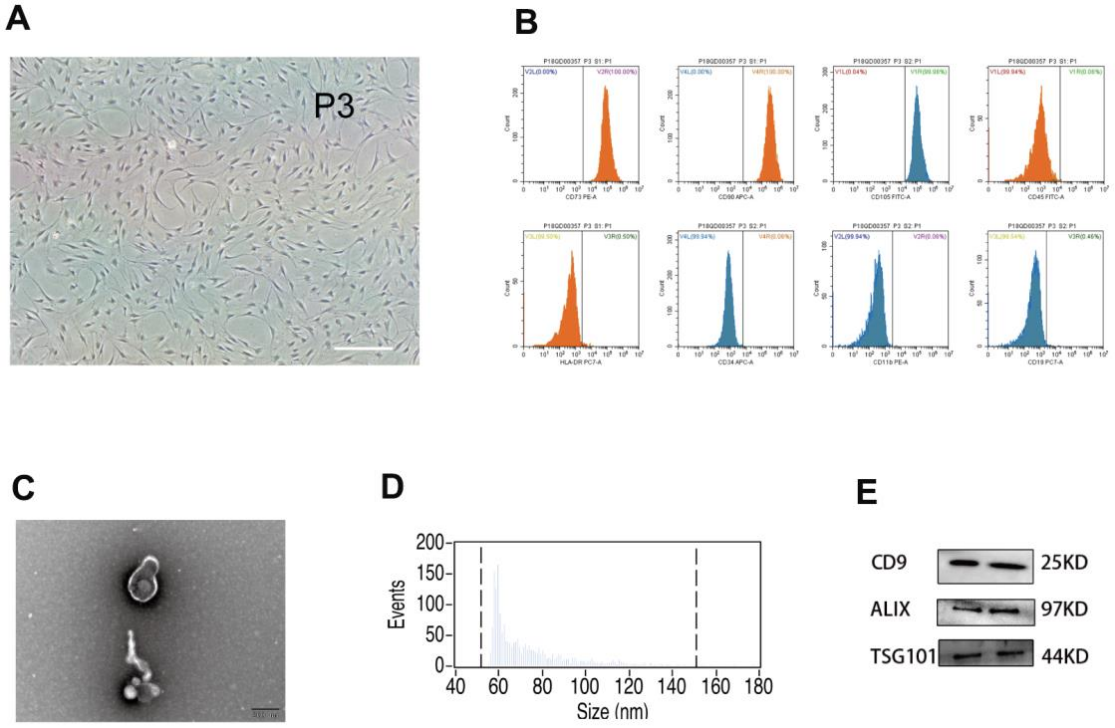
**Analysis of human umbilical mesenchymal stem cells (hUMSCs) and hUMSC-derived exosomes (hUMSC-Exos).** (**A**) Representative micrographs of cultured hUMSCs at passage 3 (P3). Scale bar: 200 μm. (**B**) Flow cytometry analysis of hUMSC CD73, CD105, CD90, CD11b, CD19, CD34, CD45, and HLA-DR expression. (**C**) Representative electron micrographs of hUMSC-Exos. Scale bar: 200 nm. (**D**) Exosome particle size and concentration. (**E**) Western blot analysis of Exos-specific markers CD9, ALIX, and TSG101. Each blot represents three independent experiments of two samples each.

*In vitro*, hUMSCs were expanded to the third and fifth generations (Figure 1A). Flow cytometry was used to characterize hUMSC surface phenotype (Figure 1B). The majority of hUMSCs were positive for expression of CD73 (100%), CD105 (99.96%), and CD90 (100%), and negative for expression of CD45 (0.06%), HLA-DR (0.50%), CD34 (0.06%), CD11b (0.06%), and CD9 (0.46%). Such results are representative of the known hUMSC phenotype, confirming hUMSC identity. After removal of dead cells and debris from the hUMSC-conditioned culture medium, secreted extracellular vesicles were harvested by differential centrifugation. Such vesicles displayed typical exosomal features, including morphology and size (30-150 nm) as detected by electron microscopy (Figure 1C) and NanoSight analysis (Figure 1D), and the presence of Exos markers CD9, Alix, and TSG101 [36] as detected by western blot (Figure 1E). Therefore, they were designated hUMSC-Exos.

### In a murine model, hUMSC-Exos alleviate ischemic stroke injury and inflammation

We examined the *in vivo* effects of hUMSC-Exos in a murine model of ischemic stroke. Mice were randomly allocated to vehicle-only (phosphate-buffered saline (PBS)) or experimental (hUMSC-Exo in PBS) groups, and interventions were intravenously administered via the tail vein four hours post-reperfusion (Figure 2A). Seventy-two hours post-reperfusion, neurological function scores were examined. Lower Bederson scale and higher grip strength test scores (Figure 2C, P < 0.05) were observed in the experimental group. After experimental animal euthanasia, brain slices were obtained to assess infarct volume via 2,3,5-triphenyltetrazolium chloride (TTC) staining, demonstrating smaller infarct size in the experimental group (Figure 2B, p < 0.05). Additionally, the area of ischemic penumbra was smaller in the experimental group (Figure 2D). Furthermore, hematoxylin and eosin (H&E) staining demonstrated lower levels of tissue edema and cell edema and fewer contracted nuclei in the experimental group (Figure 2E). Immunohistochemical detection of IL-6 and NFκB in the ischemic penumbra demonstrated significantly lower expression of these proteins in the experimental group (Figure 2F, p < 0.05). Finally, infiltration of PKH26 (red)-labeled intravenous Exos into the site of brain injury was demonstrated by fluorescence microscopy (Figure 2G). Taken together, these results suggest that circulating hUMSC-Exos infiltrate the relevant anatomical site and are protective against I/R injury after ischemic stroke, in part by decreasing levels of local neuroinflammation.

**Figure 2:**
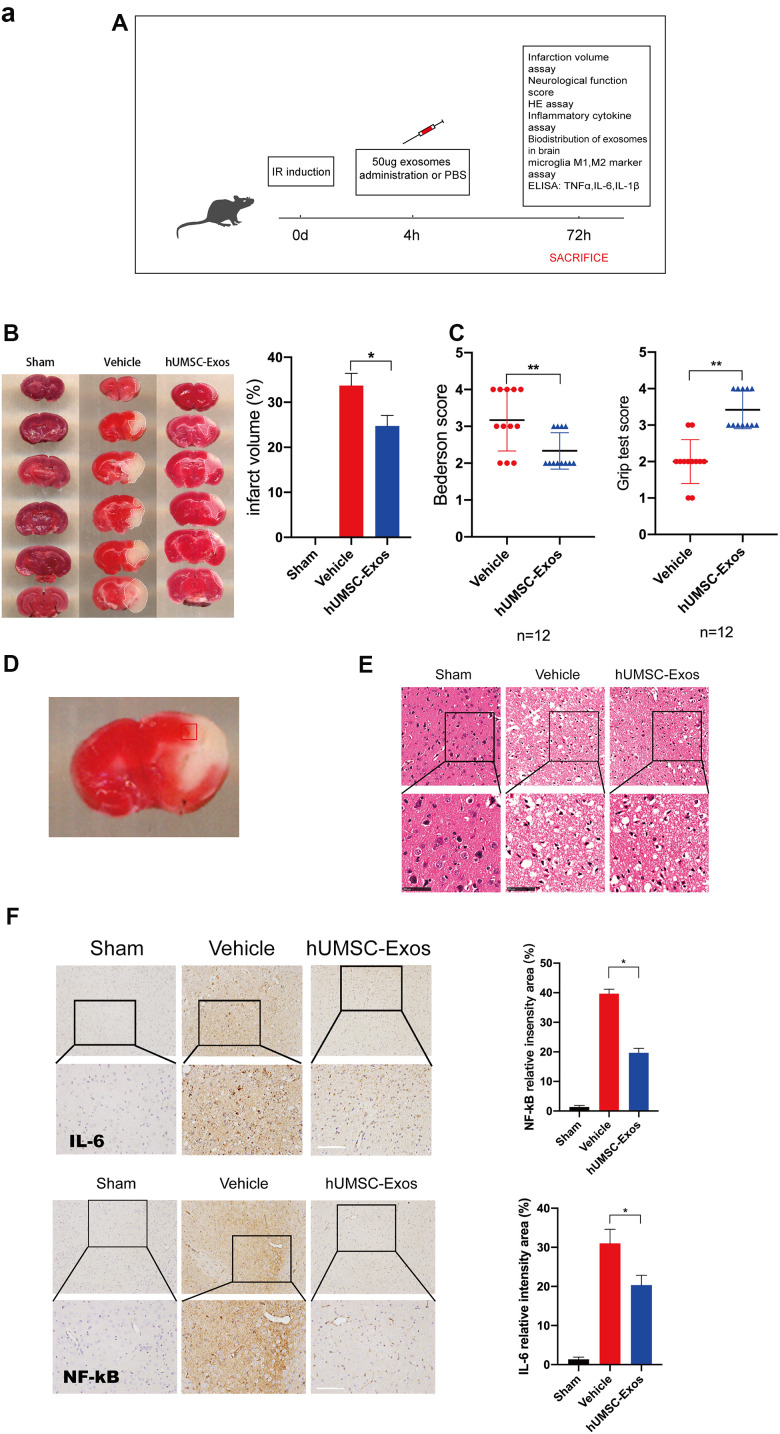
**Treatment with hUMSC-Exos attenuates microglia-mediated inflammation and neurological deficits after ischemic stroke.** (**A**) Schematic of the protocol. (**B**) Representative photomicrographs of TTC-stained tissue from the control, vehicle-only, and experimental groups, with associated infarct size as calculated using ImageJ software. Data are expressed as mean ± SEM (n = 12 per group). Significant differences are indicated (*p < 0.05). (**C**) Neurological deficit scores in the vehicle-only and experimental groups 72 hours post-reperfusion. Data are expressed as mean ± SEM (n = 12 per group). Significant differences are indicated (*p < 0.05, **p < 0.01). (**D**) The red box indicates the cerebral ischemic penumbra. (**E**) H&E staining. Scale bar: 50 μm. (**F**) Representative photomicrographs of IL-6 and NFκB in the ischemic penumbra 72 hours post-reperfusion, with associated relative intensities as calculated using ImageJ software. Scale bar: 50 μm. Data are expressed as mean ± SEM (n = 6 per group). Significant differences are indicated (*p < 0.05).

**Figure 2:**
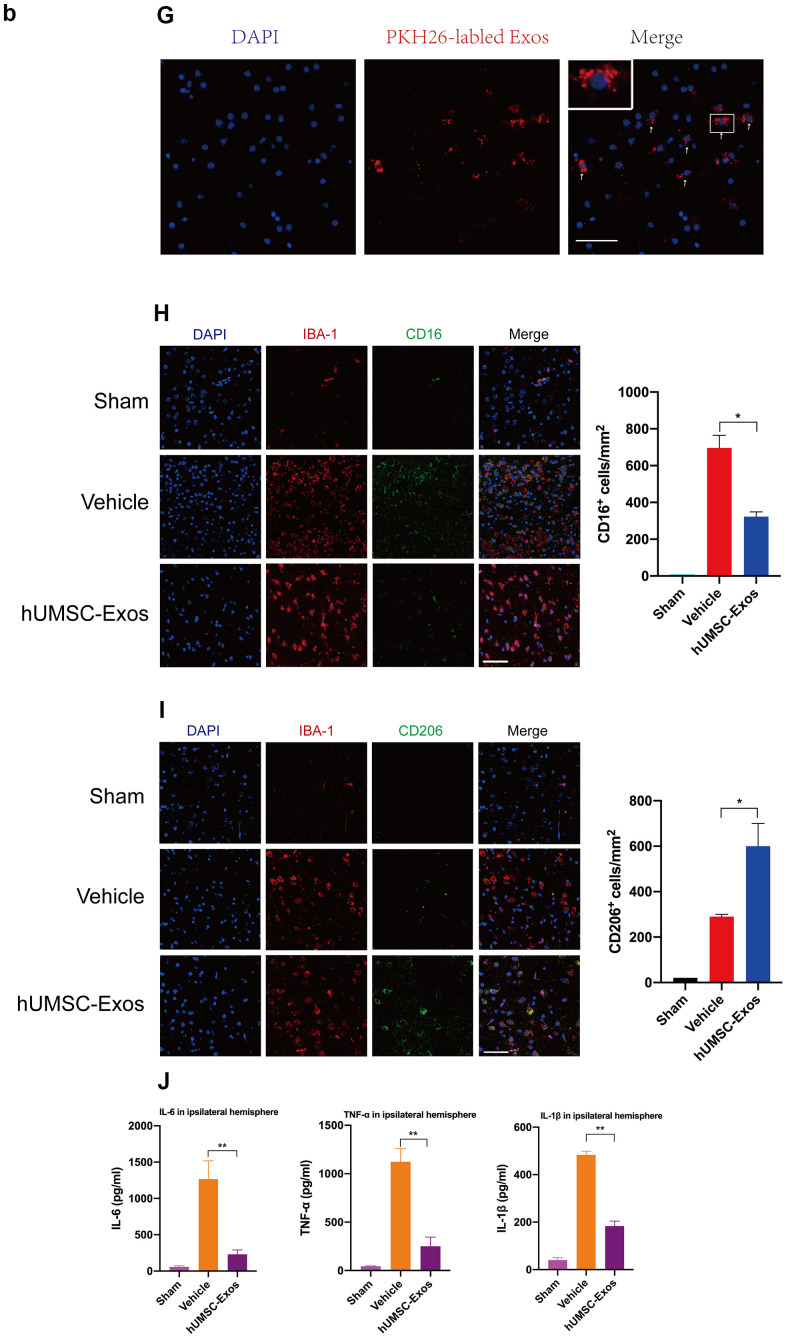
**Treatment with hUMSC-Exos attenuates microglia-mediated inflammation and neurological deficits after ischemic stroke.** (**G**) Red fluorescence indicates PKH26-labeled exosomes which have accessed the site of cerebral damage. Scale bar: 50 μm. (**H**) Microglial M1 markers IBA-1 and CD16 in the ischemic penumbra 3 days following ischemic stroke, in the control, vehicle-only, and experimental groups. Scale bar: 50 μm. Associated M1 counts are shown (**A**, **B**). (**I**) Microglial M2 markers IBA-1 and CD206 in the ischemic penumbra 3 days following ischemic stroke, in the control, vehicle-only, and experimental groups. Scale bar: 50 μm. Associated M2 counts - from the same animals in which M1 counts were determined - are shown (**C**, **D**). Significant differences are indicated (*p < 0.05). (**J**) Lower protein levels of pro-inflammatory cytokines IL-6, TNF-α, and IL-1β in the experimental group. Data are expressed as mean ± SEM (experiments were performed in triplicate). Significant differences are indicated (*p < 0.05, **p < 0.01).

We next examined the effect of hUMSC-Exos on activated microglia *in vivo*. Treatment with hUMSC-Exos markedly decreased the presence of IBA-1^+^CD16^+^ cells at 72 h post-stroke (Figure 2H, p < 0.05) but markedly increased the presence of IBA-1^+^CD206^+^ cells at 72h post-stroke (Figure 2I, p < 0.05). Since IBA-1 is a marker of brain microglia [37], CD16 is an M1 marker [38], and CD206 is an M2 marker [39], this suggests that hUMSC-Exos treatment decreased and increased, respectively, the number of M1 and M2 microglia. Expression of pro-inflammatory cytokines IL-6, TNF-α, and IL-1β was also significantly decreased in the experimental group (Figure 2J, p < 0.01). Taken together, these results suggest that hUMSC-Exos may decrease microglia-mediated neuroinflammation after ischemic stroke in mice.

### Microglial pro-inflammatory activity is also decreased by hUMSC-Exos *in vitro*

**Figure 3:**
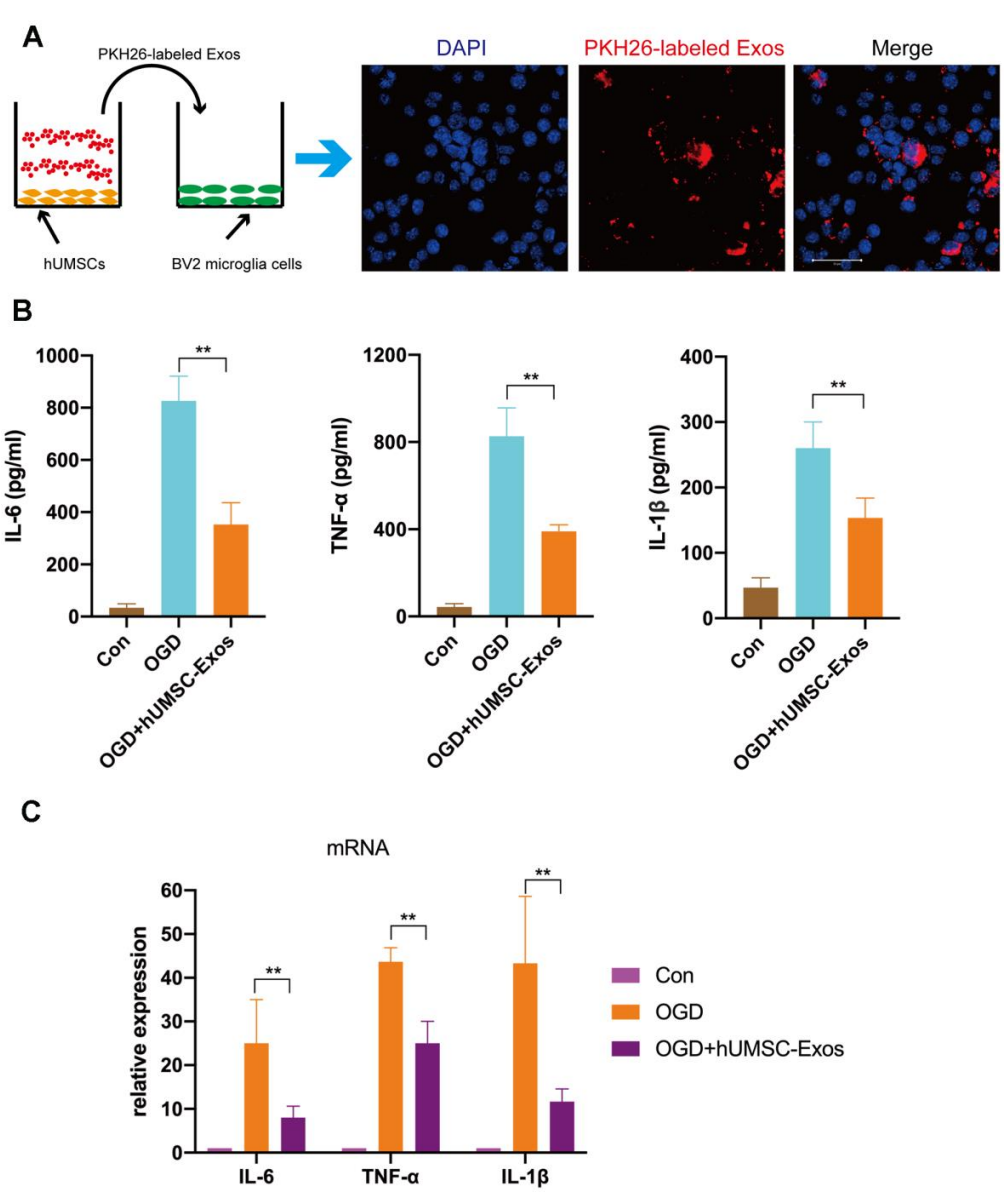
**Treatment with hUMSC-Exos reduces microglial pro-inflammatory activity *in vitro*.** (**A**) Confocal imaging demonstrating uptake of PKH-26-labeled exosomes (red) by BV2 microglia. Scale bar: 50 μm. (**B**) Lower protein levels of pro-inflammatory cytokines IL-6, TNF-α, and IL-1β in the hUMSC-Exos treatment group. (**C**) Levels of IL-6, TNF-α, and IL-1β mRNA as detected using qRT-PCR. Data are expressed as mean ± SEM (experiments were performed at least in triplicate). Significant differences are indicated (*p < 0.05, **p < 0.01).

First, we tested whether red fluorescent dye (PKH26)-labeled hUMSC-Exos are internalized during co-culture with BV2 microglia. After 6 h, microglia had efficiently internalized hUMSC-Exos as indicated by intracellular fluorescence (Figure 3A). To further validate the direct effects of hUMSC-Exo on activated microglia, the latter were cultured in serum-free medium for 6 h in a hypoxic incubator (to mimic oxygen-glucose deprivation (OGD)) prior to culture in conventional medium with or without hUMSC-Exos. After 24 h, IL-6, TNF-α, and IL-1β intracellular transcription and supernatant protein levels were determined by real-time polymerase chain reaction (RT-PCR) and enzyme-linked immunosorbent assay (ELISA), respectively. Treatment with hUMSC-Exos significantly decreased both IL-6, TNF-α, and IL-1β transcription and protein levels (Figure 3B, 3C, p < 0.01). Results suggest that hUMSC-Exos decrease microglial pro-inflammatory activity *in vitro*.

### Microglial pro-inflammatory activity is attenuated by hUMSC-Exosomal miRNAs *in vitro*

**Figure 4:**
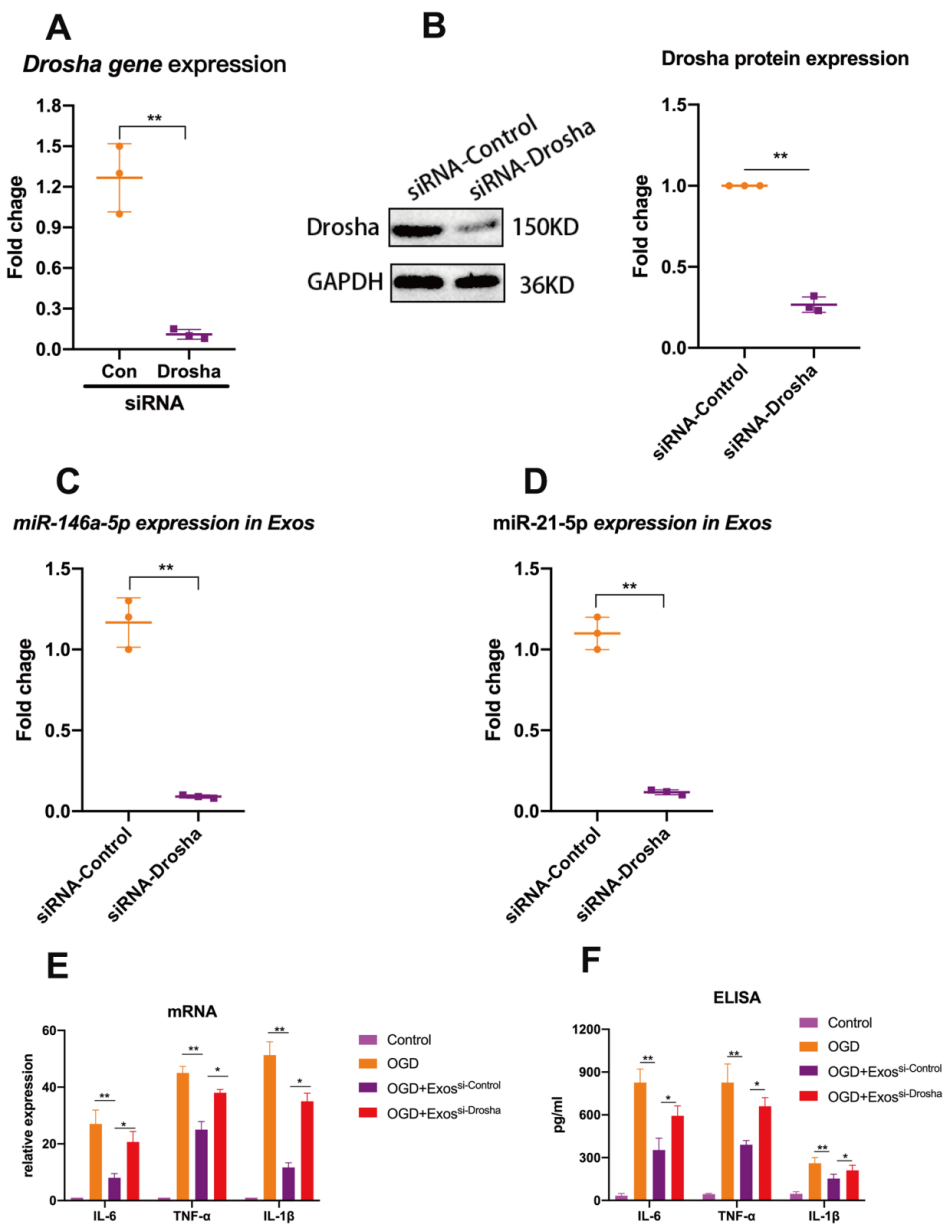
**Exosomal miRNAs are implicated in hUMSC-Exos-mediated attenuation of microglial pro-inflammatory activity.** (**A**, **B**) After 24 hours’ siRNA-Drosha transfection, hUMSC Drosha knockdown efficiency was evaluated by qPCR quantitation of Drosha mRNA and western blot-based quantitation of Drosha protein. Western blots are representative of three independent experimental replicates. (**C**, **D**) Exosomal miR-146a-5p and miR-21-5p content was significantly decreased after Drosha knockdown. (**E**) Protein levels of the pro-inflammatory cytokines IL-6, TNF-α, and IL-1β in hUMSC-Exos were decreased after Drosha knockdown. (**F**) Detection of IL-6, TNF-α, and IL-1β mRNA levels via qRT-PCR. Data are expressed as mean ± SEM. (A-F) Each experiment is representative of n = 3 per group. Significant differences are indicated (*p < 0.05, **p < 0.01).

In an attempt to elucidate at least one mechanism by which hUMSC-Exos modulate microglial activity, we investigated whether hUMSC-Exosomal miRNAs attenuate microglial pro-inflammatory activity *in vitro*. To demonstrate that miRNAs are a key functional component of these Exos, we conducted small interfering RNA (siRNA) knockdown of Drosha (an essential polymerase required for miRNA synthesis) in hUMSC (Figure 4A, 4B) in order to generate miRNA-depleted hUMSC-Exos (Figure 4C, 4D). Treatment of activated microglia with wild-type or Drosha-knockdown hUMSC-Exos demonstrated that miRNA depletion significantly weakened the anti-inflammatory effect of hUMSC-Exos (Figure 4E). Results suggest that hUMSC-Exosomal miRNAs contribute to microglial modulation.

### Specifically, hUMSC-Exosomal miR-146a-5p attenuates microglial pro-inflammatory activity *in vitro* through suppression of the IRAK1/TRAF6 signaling pathway

**Figure 5:**
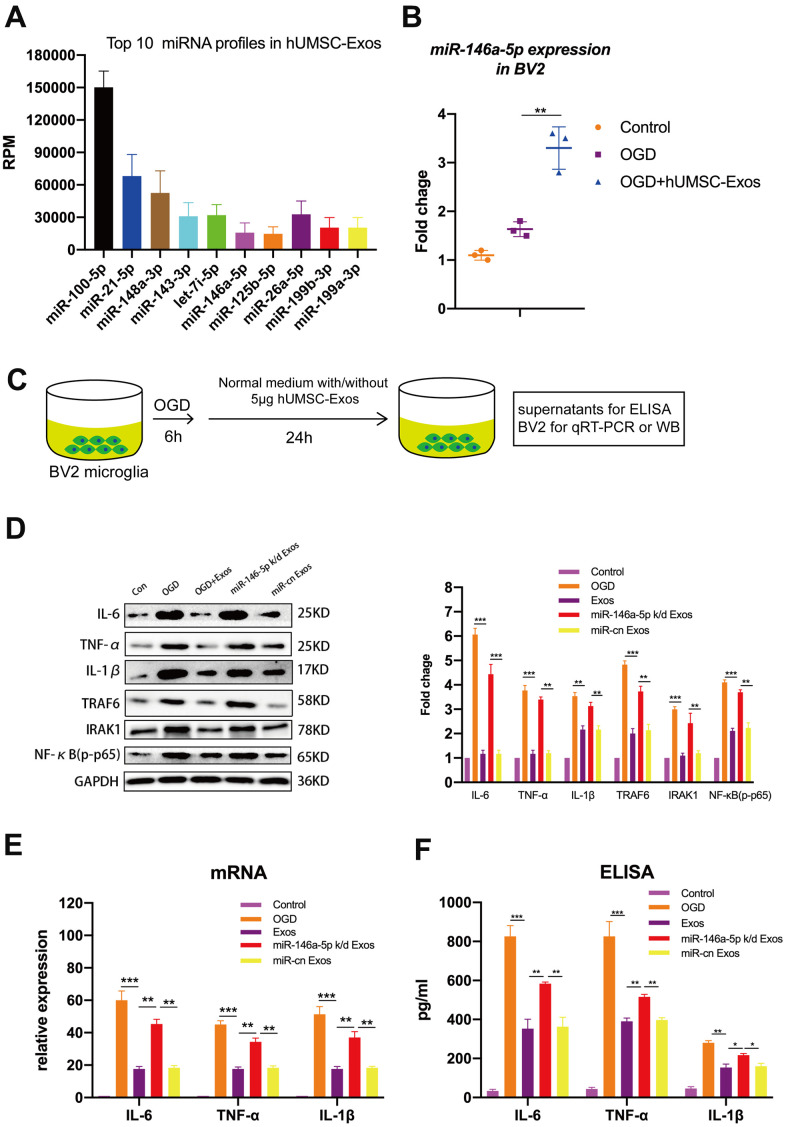
**Exosomal miR-146a-5p decreases microglial pro-inflammatory activity by suppressing the IRAK1/TRAF6 signaling pathway *in vitro*.** (**A**) Expression levels of the top ten hUMSC-Exosomal miRNAs, including MiR-146a-5p. (**B**) After post-OGD exposure to hUMSC-Exos, BV2 microglia exhibited significantly increased miR-146a-5p content. Data were normalized to levels of U6. (**C**) *In vitro* experimental scheme. (**D**) Expression of pro-inflammatory cytokines IL-6, TNF-α, and IL-1β, as well as signaling pathway IRAK1, TRAF6, and NFκB (p65) in microglia treated with wild-type versus miR-146a-5p knockdown hUMSC-Exos. (**E**) Determination of IL-6, TNF-α, and IL-1β mRNA levels via qRT-PCR. (**F**) Determination of supernatant IL-6, TNF-α, and IL-1β protein levels via ELISA. Data are expressed as mean ± SEM. (**A**–**F**) Each experiment is representative of n = 3 per group. Significant differences are indicated (*p < 0.05, **p < 0.01, ***p < 0,001).

To determine which hUMSC-Exosomal miRNAs may contribute to microglial polarization and attenuation of microglial pro-inflammatory activity, BV2 microglia were exposed to OGD with or without subsequent hUMSC-Exos treatment for 24 hours, and hUMSC-Exosomal small RNA expression analysis and deep sequencing were performed. The following formula was used to calculate corrected miRNA expression: reads per million (RPM) = (number of reads mapping to miRNA/number of clean reads) *10^6^. To the best of our knowledge, this is the first study to report sequencing of hUMSC-Exos-derived miRNAs. The analysis revealed that several hundred miRNA species are present within hUMSC-Exos. Among the top ten miRNAs identified as significantly differentially expressed between hUMSC-Exos treated and untreated microglia (Figure 5A), miR-146a-5p was higher in the treatment group and is known to modulate inflammation [40]. As demonstrated by PCR, treatment with hUMSC-Exos significantly increases BV2 microglial miR-146a-5p content (Figure 5B). Results suggest that hUMSC-Exosomal miR-146a-5p is internalized by microglia (or that hUMSC-Exos induce microglial miR-146a-5p expression) and may contribute to modulating inflammation. The *in vitro* experimental scheme is pictured (Figure 5C). Western blot analysis demonstrated decreased IL-6, TNF-α, and IL-1β, as well as IRAK1/TRAF6 signaling pathway member IRAK1, TRAF6, and NFκB (p65) protein expression after hUMSC-Exos treatment. Furthermore, expression of IL-6, TNF-α, and IL-1β was higher after treatment with miR-146a-5p knockdown hUMSC-Exos (Figure 5D–5F). Results suggest that hUMSC-Exosomal miR-145-5p contributes to modulating OGD-induced microglial pro-inflammatory activity via suppression of the IRAK1/TRAF6 signaling pathway.

### Microglia-mediated neuroinflammation and neural deficits resulting from ischemic stroke are attenuated by hUMSC-Exosomal miR-146a-5p in mice

**Figure 6:**
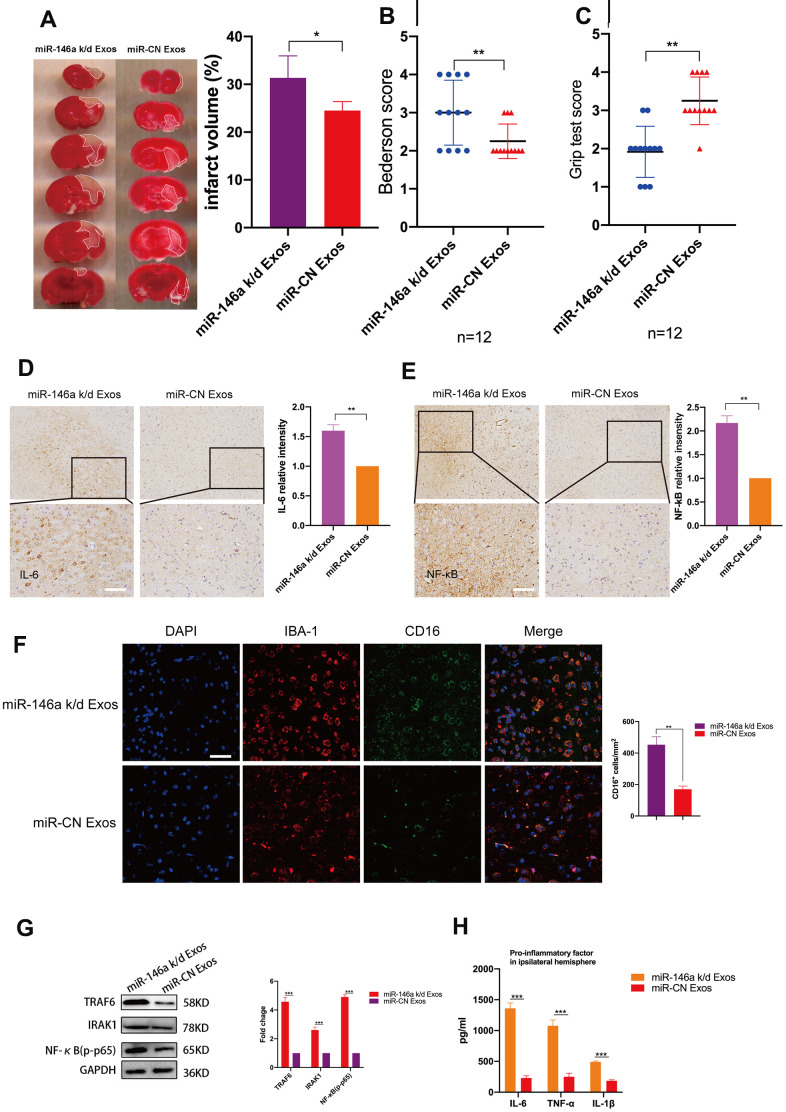
**Treatment with hUMSC-Exos decreases neuroinflammation and is neuroprotective by down-regulating IRAK1/TRAF6 signaling pathway activity *in vivo*.** (**A**) Representative photomicrographs of TTC-stained tissue from wild-type versus miR-146a-5p knockdown hUMSC-Exos groups, with infarct size as calculated using ImageJ software. Data are expressed as mean ± SEM (n = 6 per group). Significant differences are indicated (*p < 0.05). (**B**, **C**) Neurological deficit scores in vehicle-only versus experimental groups at 72 hours post-reperfusion. Data are expressed as mean ± SEM (n = 12 per group). Significant differences are indicated (*p < 0.05, **p < 0.01). (**D**, **E**) Representative photomicrographs of IL-6 and NFκB in the ischemic penumbra 72 hours post-reperfusion, with associated relative intensities as calculated using ImageJ software. Scale bar: 50 μm. Data are expressed as mean ± SEM (n = 6 per group). Significant differences are indicated (*p < 0.05). (**F**) Microglial M1 markers IBA-1 and CD16 in the ischemic penumbra 3 days following ischemic stroke. (**G**) Expression of signaling pathway IRAK1, TRAF6, and NFκB (p65) in the wild-type versus miR-146a-5p knockdown groups. (**H**) Determination of IL-6, TNF- α, and IL-1β protein levels via ELISA. Data are expressed as mean ± SEM (experiments were performed in triplicate). Significant differences are indicated (*p < 0.05, **p < 0.01, ***p < 0,001).

To investigate whether hUMSC-Exosomal miR-146a-5p exerts neuroprotective effects in the ischemic mouse brain via IRAK1/TRAF6 signaling pathway modulation, both wild-type and miR-146a-5p knockdown hUMSC-Exos were administered in a murine ischemic stroke model. Relative to treatment with miR-146a-5p knockdown hUMSC-Exos, treatment with wild-type hUMSC-Exos significantly reduced infarct volume three days post-ischemia (Figure 6A) and resulted in lower Bederson scale scores and higher grip strength test scores (Figure 6B, 6C). Results suggest that hUMSC-Exosomal miR-145a-5p may attenuate I/R damage and neural deficits after ischemic stroke in a murine model.

Immunohistochemistry demonstrated that levels of IL-6 and NFκB in the ischemic penumbra were significantly lower in the group treated with wild-type hUMSC-Exos (Figure 6D, 6E, p < 0.01). Furthermore, immunofluorescent labeling demonstrated that significantly fewer microglia were activated in the group treated with wild-type hUMSC-Exos (Figure 6F, p < 0.01). Western blots demonstrated that expression of IRAK1/TRAF6 signaling pathway proteins IRAK1, TRAF6, and NFκB (p65) was significantly decreased in the group treated with wild-type hUMSC-Exos (Figure 6G, p < 0.001). Finally, levels of IL-6, TNF-α, and IL-1β were significantly reduced in the group treated with wild-type hUMSC-Exos (Figure 6H, p < 0.001). Results suggest that hUMSC-Exosomal miR-146a-5p may attenuate microglia-mediated neuroinflammation after ischemic stroke in a murine model.

## DISCUSSION

*In vivo*, OGD activates microglial-mediated inflammatory response [41]. During the acute period after stroke, microglia secrete pro-inflammatory cytokines IL-6, TNF-α, and IL-1β [38], which can induce secondary cytotoxicity. Studies have proven that decreasing microglia-mediated neuroinflammation is beneficial during stroke recovery [13, 17]. Although MSC transplantation is neuroprotective after both traumatic brain injury and stroke, at least in part via modulating microglia-mediated neuroinflammation, mechanisms of the latter remain incompletely understood. However, since over 99% of transplanted MSCs become entrapped in the pulmonary vasculature without impeding therapeutic effect, mechanisms likely involve distally acting MSC-produced paracrine factors which may hold promise as cell-free therapies. Nearly all cell types secrete exosomes, which are important mediators of cellular communication.

One important group of Exos cargo molecules is miRNAs (short non-coding RNAs that inhibit target gene expression by directly binding their mRNAs) [42]. Prior studies report that Exos play an important role in transmitting miRNAs between cells [43–46], via interstitial fluid and circulation delivering biologically active miRNAs to both neighboring and distant cells [47]. For example, adipose tissue constitutes an important source of circulating exosomal miRNAs that serve as a previously unrecognized form of adipokine to regulate gene expression in distant tissues [48], adipose tissue-resident macrophage-derived exosomal miRNAs modulate insulin sensitivity [43], and exosomal transfer of miR-181b from cardiosphere-derived cells (CDCs) into macrophages reduces PKCδ transcription (a mechanism underlying the post-reperfusion cardioprotective effects of CDCs) [49]. The present study investigated the potential therapeutic role hUMSC-Exos in ameliorating I/R injury.

Results suggest that in a murine model of ischemic stroke, quality-controlled hUMSC-Exos delivered intravenously four hours post-reperfusion are able to traverse the blood-brain barrier to access the site of ischemic injury and are then taken up by local microglia, in which exosome-derived miR-146a-5p inhibits IRAK1/TRAF6 signaling pathway-mediated NFκB activation and consequent M1 polarization and production of potent pro-inflammatory cytokines (instead favoring M2 polarization), ultimately resulting in decreased: I/R-induced tissue edema, cell death, extent of the ischemic infarct and penumbra, and functional motor deficits.

This is consistent with prior studies which have demonstrated that immunofluorescently labeled Exos are detectable both extracellularly and intracellularly at sites of brain injury [50] and are taken up by recipient cells [50, 51]. It has also previously been demonstrated that MSC-derived Exos exert powerful effects in the context of ischemic stroke, for example ameliorating inflammation-induced astrocyte alterations [52]. Regarding mechanisms, pro-neurogenic effects of UMSC-Exos may be partially attributable to histone deacetylase 6 (HDAC6) inhibition by exosomal miR-26a [53]. Furthermore, it is known that without miRNA-184 and -210, MSC-derived extracellular vesicles lose the ability to promote neurogenesis and angiogenesis [54]. Although it is difficult to completely exclude the effects of other exosomal cargo molecule groups on microglia in the present study, miRNAs are considered a key functional element. The miRNA miR-146a-5p is a well-known anti-inflammatory molecule with a key role in inflammatory disorders [55–57]. The receptor proteins IRAK1 and TRAF6 are abundant in the cytoplasm and nucleus of various cell types [58]. They are largely involved in Toll-like receptor (TLR)-initiated pathways, leading to expression of pro-inflammatory mediators [59]. Overexpression of IRAK1 and TRAF6 can activate NFκB [60], a transcription factor which is a key activator of pro-inflammatory gene expression programs [57]. Through binding the 3′UTR of the mRNAs encoding IRAK1 and TRAF6, miR-146a-5p down-modulates inflammatory responses [61, 62].

Apart from recommending follow-up research into the therapeutic potential of cell-free miR-146a-5p in the context of I/R injury and inflammatory disorders, results raise a number of interesting theoretical questions. For example, it is unknown whether hUMSC-Exosomal cargo is also perhaps involved in regulating glial ion transporters (which are involved in the Warburg effect, glial activation, neuroinflammation, and neuronal damage during glioma [63]). Additionally, might other miRNAs impact microglial function after ischemic stroke? Might hUMSC-Exos also impact the function of other local cell types (e.g. neurons, astrocytes, and/or oligodendrocytes) after ischemic stroke? Finally, by which mechanisms might such effects occur?

## CONCLUSIONS

**Figure 7:**
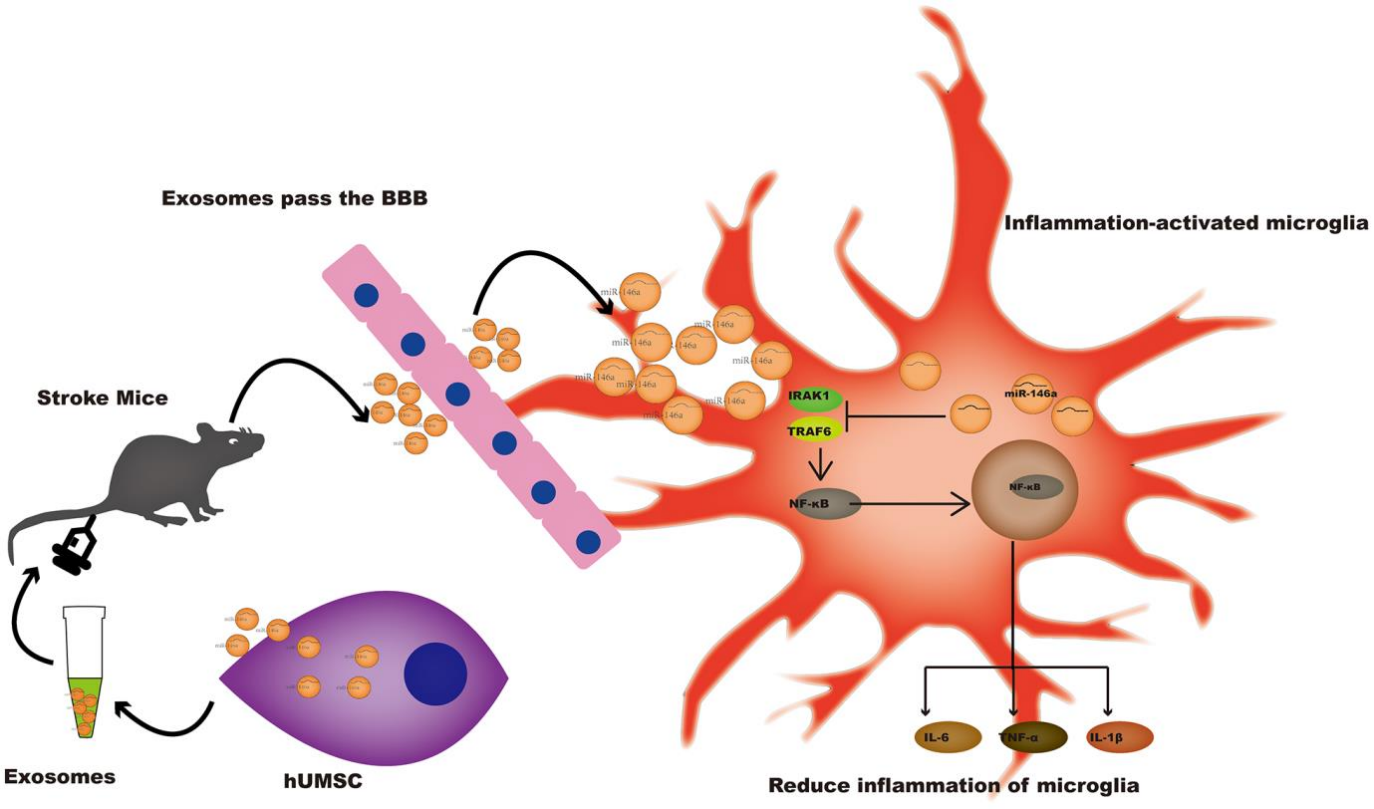
**A potential mechanism contributing to the hUMSC-Exos-induced decrease in microglia-mediated neuroinflammation after ischemic stroke.** After injection of hUMSC-Exos into the tail vein of the murine ischemic stroke model, they traversed the blood-brain barrier and were internalized by microglia at the site of cerebral injury. Exosomal miR-146a-5p may decrease microglia-mediated neuroinflammation by suppressing the IRAK1/TRAF6 signaling pathway.

In conclusion, hUMSC-Exos and or miR-146a-5p represent novel therapeutic options for the improvement of outcomes after ischemic stroke (Figure 7), warranting further investigation.

## MATERIALS AND METHODS

### Animals

Experimental animals (C57BL/6 mice aged 8 weeks, weighing 20-30 g each) were purchased from the Animal Experiment Center of Southern Medical University (Guangzhou, China), fed a standard laboratory diet with *ad libitum* access to food and water, and maintained at a temperature of 22 ± 1° C and a humidity of 65–70 % in a controlled room with a 12 h light–dark cycle. All experimental procedures were approved by the Southern Medical University Ethics Committee and performed in accordance with the National Institutes of Health Guidelines for the Care and Use of Laboratory Animals.

### Murine model of ischemic stroke

As described previously [64], mice were anesthetized via intraperitoneal injection (35 mg/kg sodium pentobarbital). Transient middle cerebral artery occlusion (MCAO) was produced by advancing a 4-0 nylon monofilament (0.23–0.25 mm) (Yushun Bio Technology Co. Ltd., China) via the left common carotid artery to occlude the middle cerebral artery for 60 minutes, prior to filament withdrawal (reperfusion). Success of blood-flow occlusion and restoration was verified using a laser Doppler flowmeter (Moor LAB, Moor Instruments, Devon, UK). During the MCAO procedure, head temperature was maintained at 36° C. At four hours post-reperfusion, 250 μL PBS with or without 50 μg hUMSC-Exos was injected into the tail vein (experimental and vehicle-only groups). Mice in a third (control) group received no injection.

### Evaluation of motor function

At 24 hours post-reperfusion, global neurological and motor function was assessed (Table 1) using a modified Bederson Scale and grip strength evaluation [65] by a researcher blinded to group allocation. For grip strength evaluation, taut string (50 cm) was suspended between two vertical supports at a height of 40 cm. Each mouse was placed midway on the string and rated as shown in Table 1.

**Table 1:** Neurological function score.

**Score**	**A modified Bederson score**	**Score**	**The grip test**
0	No deficit.	0	Falls off.
1	Forelimb flexion.	1	Hangs onto string by one or both forepaws.
2	As for 1, plus decreased resistance to lateral push.	2	As for 1, and attempts to climb onto string.
3	Unidirectional circling.	3	Hangs onto string by one or both forepaws plus one or both hind paws.
4	Longitudinal spinning or seizure activity.	4	Hangs onto string by fore and hind paws plus tail wrapped around string.
5	No movement.	5	Escape (to the supports).

### Evaluation of cerebral infarct volume

At 72 hours post-reperfusion, mice were euthanized. Thereafter, the brain was removed for coronal sectioning. Infarct size was measured using 2% 2,3,5-triphenyltetrazolium chloride (TTC) staining in conjunction with microscopy. Infarct volume was evaluated by a blinded observer using ImageJ software version 1.61 (National Institutes of Health, Bethesda, MD, USA).

### H&E staining

Fresh brain tissues were fixed using 4% PFA (pH 7.4), gradually dehydrated, embedded in paraffin, cut into 4-μm-thick sections using a microtome, and stained with H&E to visualize cellular structures by microscopy.

### Immunofluorescent staining

Four-micron-thick coronal brain sections were deparaffinized in xylene, rehydrated via an alcohol gradient, and washed with PBS (0.01 M, pH 7.4). Sections were blocked using 5% bovine serum albumin (BSA) for 60 min at room temperature prior to overnight incubation at 4° C with the following primary antibodies: goat anti-IBA-1 (1:200 dilution; ab48004, Abcam), rabbit anti-CD16 (1:200 dilution; ab252908, Abcam), and goat anti-CD206 (1:200 dilution, R&D Systems). Automated image analysis was performed using ImageJ software version 1.61 (National Institutes of Health, Bethesda, MD, USA). Cell numbers were calculated per square millimeter from three random microscopic fields (200 × magnification) on three sections (a total of nine images) (n = 6 animals per group). All counts were performed in a blinded manner.

### Cell types and cell culture methods

Human umbilical cords were provided by Guangzhou Saliai Stem Cell Science and Technology Co. Ltd. for hUMSC isolation. Thereafter, hUMSCs were cultured in complete Dulbecco’s modified Eagle’s medium (DMEM)/F12 (Gibco) supplemented with 10% fetal bovine serum (FBS) (Gibco, Australia), and cells between the 3^rd^ and 5^th^ generations were used for subsequent experiments. The BV2 murine microglial cell line was obtained from Xiehe Medical University (Beijing, China). Microglia were cultured in DMEM supplemented with 10% heat-inactivated FBS, 100 μg/mL streptomycin, and 100 U/mL penicillin (HyClone).

### Exosome isolation, characterization, and treatment

After hUMSCs were cultured in DMEM/F12 medium supplemented with 10% (FBS), culture supernatants containing Exos were centrifuged at 2,000 x g for 20 min at "4° C, followed by 10, 000 x g for 30 min at 4° C, and the supernatant was passed through a 0.22 μm filter (Millipore), to pellet and exclude contaminating dead cells and debris. Thereafter, the filtrate was subjected to ultracentrifugation at 110,000 g for 70 min at 4° C to pellet Exos. Pellets were washed with PBS, followed by ultracentrifugation at 110,000 × g for 70 min at 4° C to re-pellet Exos, which were then resuspended in PBS. The bicinchoninic acid (BCA) Protein Assay kit (KeyGEN BioTECH) was used to estimate Exos concentration. For transmission electron microscopy (JEM-1200EX, JEOL Ltd.), 5-10 μl of each sample was added to a copper mesh and precipitated for 3 min. Remaining liquid was carefully pipetted from the filter paper edge. Thereafter, filter paper was rinsed with PBS and phosphotungstic acid was used for negative staining prior to drying at room temperature for 2 min and imaging (operating voltage: 80-120 kV).

### Determining Exos internalization by microglia via fluorescence microscopy

Exos were labeled using the red fluorescent membrane dye PKH67 (Sigma). Labeled Exos were washed by resuspension in 10 mL PBS, pelleted by ultracentrifugation as described above, and finally resuspended in 100 μl PBS. For cell treatment, a volume of suspension corresponding to 2 μg of Exos was added to 2×10^5^ recipient cells prior to 24 hours of incubation.

### *In vitro* OGD and “reperfusion” model

An anaerobic chamber containing 95% N_2_ and 5% CO_2_ was used in conjunction with deoxygenated glucose-free DMEM (Gibco) was used to simulate microglial OGD. After 6 hours, culture medium was replaced with maintenance medium, and cells were moved to a regular incubator to recover for 24 hours.

### RT-PCR

Total RNA was extracted from cells and brain tissues using Trizol reagent (Life Technologies) and from Exos using an Exosome RNA Purification Kit (EZB-exo-RN1), both according to the manufacturer’s instructions. Reverse transcription was performed using the PrimeScript RT reagent Kit (RR037A, Takara Bio Inc., Shiga, Japan). Real-time PCR was conducted using SYBR Green PCR Master Mix (Applied TaKaRa, Otsu, Shiga, Japan) in conjunction with an Applied Biosystems 7500 Fast Real-Time PCR System (Applied Biosystems, Foster City, CA, USA). Sequences of all primers are provided (Table 2).

**Table 2:** Primers used for real-time PCR.

**Genes**	**Primer sequences**
IL-6	FORWARD ACTTCCATCCAGTTGCCTTCTTGG
	REVERSE TTAAGCCTCCGACTTGTGAAGTGG
TNF-α	FORWARD GCGACGTGGAACTGGCAGAAG
	REVERSE GCCACAAGCAGGAATGAGAAGAGG
IL-1β	FORWARD ACTTCCATCCAGTTGCCTTCTTGG
	REVERSE TGCTCATGTCCTCATCCTGGAAGG
DROSHA	FORWARD AAGGCAAGACGCACAGGAATTAGG
	REVERSE TCTGCCAGCATTGTTGGTCATAGG
Arg-1	FORWARD CATATCTGCCAAAGACATCGTG
	REVERSE GACATCAAAGCTCAGGTGAATC
miR-21-5p	FORWARD GCGCGTAGCTTATCAGACTGA
	REVERSE AGTGCAGGGTCCGAGGTATT
miR-146a-5p	FORWARD CGCGTGAGAACTGAATTCCA
	REVERSE AGTGCAGGGTCCGAGGTATT
U6	FORWARD CTCGCTTCGGCAGCACA
	REVERSE AACGCTTCACGAATTGCGT

### Quantitation of supernatant cytokines by ELISA

Concentrations (pg/mL) of IL-6, TNF-α, and IL-1β in BV2 microglia culture supernatants and damaged cerebral tissue were determined via ELISA (kits from R&D Systems, Minneapolis, MN, USA), performed as per manufacturer instructions. Briefly, standards and samples were added to a 96-well ELISA plate pre-coated with biotinylated anti-IL-6, anti-TNF-α, and anti-IL-1β. Unbound substances were washed away, and enzyme-linked polyclonal antibodies specific for IL-6, TNF-α, and IL-1β were added to the corresponding wells. Plates were incubated for 2 hours, washed four times, and enzyme substrate was added prior to 30 min incubation. Color development was terminated using stop solution and absorbance at 450 nm was determined using a microplate reader. The concentration of each sample was calculated from a standard curve prepared using the cytokine standards. Each experiment was performed in triplicate.

### Transfection with siRNA

In order to decrease miRNA synthesis, siRNA-Drosha (Ribobio) was transfected into recipient hUMSC using riboFECTTMCP Reagent (Ribobio). After 24 hours, transfection efficiencies were evaluated via qPCR or western blots.

### Western blots

Tissues, cells, and Exos were lysed using RIPA buffer (KeyGEN BioTECH), followed by protein quantitation using a Bradford Protein Assay kit (KeyGEN BioTECH). Briefly, lysates were subjected to SDS–PAGE and transferred onto PVDF membranes (Millipore). Membranes were incubated overnight at 4° C with primary antibodies specific for the following proteins, as required by each experiment: TSG101 (1:1000 dilution; ab125011, Abcam), CD9 (1:1000 dilution; ab92726, Abcam), ALIX (1:1000 dilution; ab117600, Abcam), IRAK1 (1:1000 dilution; #511166, ZEN BIO), TRAF6 (1:1000 dilution; #380803, ZEN BIO), NFκB (p65) (1:1000 dilution; ab76302, Abcam), GAPDH (1:5000 dilution; 60004-1-Ig, Proteintech), IL-6 (1:1000 dilution; ab6672, Abcam), TNF-α (1:1000 dilution; Ab8348, Abcam), IL-1β (1:1000 dilution; 12242S, CST), and Drosha (1:1000 dilution; ab183732, Abcam). Thereafter, membranes were incubated with the relevant horseradish peroxidase (HRP)-conjugated secondary antibody to visualize protein spots using an enhanced chemiluminescence (ECL) kit (Thermo Scientific). Housekeeping protein GAPDH was selected as an internal control. Each experiment was conducted in triplicate.

### Microarray analysis

The miRNA content of hUMSC-Exos (n = 3) total RNA was profiled via small RNA deep sequencing analysis (Illumina). Library preparation and miRNA sequencing were performed by Ribobio (Guangzhou, China). Briefly, total RNA samples were fractionated and only small RNAs (18-30 nucleotides in length) were used for library preparation. After amplification by PCR, products were sequenced using the Illumina HiSeq 2500 platform.

### Knockdown of miR-146a-5p via lentiviral vector transduction

Lentiviruses expressing miR-146a-5p inhibitor were used to transduce hUMSCs at a multiplicity of infection (MOI) of 200 particles/cell. The procedure was performed in 24-well plates in DMEM (HyClone), in a 5% CO_2_ incubator at 37° C for three days. Successful transduction was confirmed by assessing hUMSC and hUMSC-Exos miR-146a-5p content via RT-PCR. No-load shRNA lentivirus was used as a control.

### Statistical analysis

All data are expressed as mean ± standard error (SE). Differences between two groups were analyzed by Student’s t-test (two-tailed), while differences between multiple groups were analyzed by one-way ANOVA in conjunction with the Bonferroni/Dunn *post hoc* test. A p-value < 0.05 was considered statistically significant. All statistical analyses were carried out using GraphPad Prism 8 software.

### Ethics approval

All experimental procedures were approved by the Ethics Committee of Southern Medical University and performed in accordance with the National Institutes of Health Guidelines for the Care and Use of Laboratory Animals.
